# Combined Deletion of miR-27a and miR-27b Enhances Protection Against Diet-Induced Obesity

**DOI:** 10.1101/2025.07.21.666011

**Published:** 2025-07-25

**Authors:** Devesh Kesharwani, Michele Karolak, Chad C. Doucette, Eric R. Loomis, Su Su, Aaron C. Brown

**Affiliations:** 1- Center for Molecular Medicine, MaineHealth Institute for Research, 81 Research Drive, Scarborough, ME 04074; 2- School of Biomedical Sciences and Engineering, The University of Maine, Orono, Maine 04469; 3- Tufts University School of Medicine, 145 Harrison Ave, Boston, MA 02111

**Keywords:** Beige adipocytes, subcutaneous adipose tissue, thermogenesis, miR-27a, miR-27b, obesity, Biological Sciences, Physiology

## Abstract

Obesity impairs adipose tissue thermogenesis, contributing to metabolic dysfunction. Here, we identify miR-27a and miR-27b as cooperative regulators of adipose tissue thermogenesis and adipogenic programming in the context of diet-induced obesity (DIO). Intervention of high-fat diet (HFD) to mice suppressed the expression of *Ucp1, Ppary*, and *Pgc1a* in inguinal white adipose tissue (WAT), which correlated with the increased expression of miR-27a/b. Using global knockout models for miR-27a and miR-27b, we identified that combined deletion of both miRNAs (double knockout, DKO) synergistically enhances *Ucp1* expression, mitochondrial heat production and browning of WAT. DKO mice displayed improved glucose, insulin sensitivity and reduced adiposity under HFD conditions and outperformed single knockouts. *In-vitro* and *ex-vivo* analysis confirmed an increase in thermogenic gene expression and reduced lipid accumulation in DKO adipocytes. Collectively, our findings reveal that miR-27a/b cooperatively suppresses adipose thermogenesis and promotes metabolic dysfunction under obesogenic conditions. Targeting the miR-27a/b axis may offer a novel therapeutic approach to enhance energy expenditure and combat obesity-related metabolic diseases.

## Introduction

Obesity has emerged as a major global health crisis, posing significant challenges to both healthcare systems and economic development^1^. The rising prevalence of obesity is closely linked to an increased risk of various metabolic disorders, including type 2 diabetes, cardiovascular diseases, and metabolic dysfunction-associated steatotic liver disease (MASLD), as well as heightened susceptibility to several forms of cancer^2–5^. The pathogenesis of obesity is multifactorial, driven by a complex interplay of genetic predisposition, environmental influences, and epigenetic regulators. These factors collectively disrupt the tightly regulated balance of energy homeostasis by affecting appetite control, nutrient absorption, and energy expenditure^6, 7^.

Adipose tissue is a key regulator of systemic energy homeostasis, with white adipose tissue (WAT) primarily responsible for energy storage, and brown adipose tissue (BAT) specialized in energy dissipation through thermogenesis^8^. In obesity, excessive expansion and functional impairment of WAT contribute to metabolic dysfunction. Enhancing energy expenditure by activating thermogenesis within adipose depots has emerged as a promising therapeutic strategy^9^. Central to this process is uncoupling protein 1 (UCP1), which facilitates the conversion of chemical energy into heat in brown adipocytes and inducible beige (brite) adipocytes within WAT^10^. Therefore, elucidating the molecular pathways that regulate UCP1 expression and promote the browning of WAT is essential for the development of novel interventions aimed at combating obesity^11^.

MicroRNAs (miRNAs) are critical post-transcriptional regulators of gene expression and play pivotal roles in diverse physiological processes, including adipogenesis and thermogenesis^8, 11^, offering novel insights into the treatment and regulation of metabolic diseases. Several miRNAs, such as miR-328, miR-378, miR-455, miR-30, and miR-32, have been shown to promote brown adipocyte differentiation and thermogenesis^12–16^. In contrast, miR-34a, miR-27a, miR-27b, miR-155, and miR-133 act as inhibitors of brown adipogenesis^17–20^. Among these, the miR-27 family, comprising miR-27a and miR-27b, has emerged as a key negative regulator of adipogenesis and thermogenesis^21^. Both isoforms are downregulated during adipocyte differentiation and function as potent inhibitors by targeting transcriptional coactivators and regulators.

Inhibition of miR-27 in cultured beige adipocytes derived from inguinal WAT significantly upregulates thermogenic genes, including *Ucp1, Prdm16*, and *Pgc1α*, whereas overexpression of miR-27 suppresses their expression^22^. Mechanistically, miR-27b directly binds to the 3’-untranslated regions (3’-UTRs) of *Pparγ* and *Prdm16*, thereby inhibiting adipocyte differentiation and thermogenic programming^23, 24^. Moreover, expression levels of miR-27a and miR-27b are elevated in the WAT of high-fat diet-induced obese mice, highlighting their potential role in suppressing thermogenesis and contributing to the development of obesity^24, 25^. Conversely, the inhibition of miR-27b using antagomirs enhances thermogenic gene expression and confers resistance to obesity, underscoring its therapeutic potential.

Our study investigates the distinct and overlapping roles of miR-27a and miR-27b in regulating thermogenesis and the development of obesity, utilizing newly created knockout mice. While prior research has largely focused on individual isoforms or their roles in adipogenesis, we directly compare the effects of single and combined deletion of miR-27a and miR-27b. Our data demonstrate that both isoforms contribute significantly to the regulation of thermogenic capacity. Notably, global double knockout (DKO) of miR-27a and miR-27b leads to a markedly enhanced thermogenic phenotype compared to single knockouts. DKO mice exhibit significantly increased *Ucp1* expression, enhanced browning of white adipose tissue (WAT), and elevated energy expenditure. These molecular and physiological changes result in a striking resistance to high-fat diet (HFD)-induced obesity in DKO mice, exceeding the protective effects observed in mice lacking only miR-27a or miR-27b.

Our findings suggest that simultaneous ablation of both isoforms is required to fully unleash *Ucp1*-dependent thermogenesis and confer maximal metabolic protection. These results underscore the miR-27 family as a critical suppressor of thermogenic programming and a promising therapeutic target for the treatment of obesity.

## Results

### Diet-Induced Obesity Impairs Adipose Tissue Thermogenic and Adipogenic Gene Expression via miR-27a/b Modulation:

**Figure 1:**
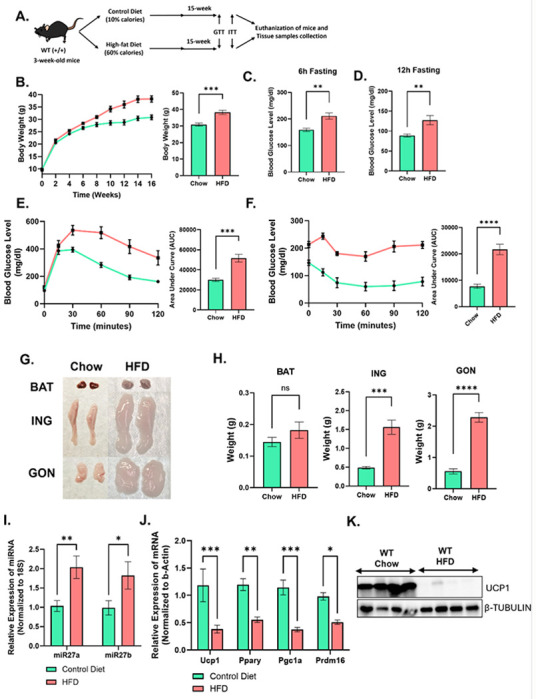
Diet-Induced Obesity Impairs Adipose Tissue Thermogenic and Adipogenic Gene Expression. **(A)** Schematic representation for induction of diet-induced obesity in wildtype mice. **(B)** Body weight of mice during and at end of diet intervention (n=7). **(C and D)** Fasting blood glucose level of mice after 16-weeks of diet. Glucose **(E)** and insulin **(F)** tolerance test were performed after the 16-weeks of HFD (n=7). **(G)** Representative appearance of brown adipose tissue (BAT), Inguinal (subcutaneous) adipose tissue (ING) and gonadal adipose tissue (GON) at the end of study. **(H)** Weight of BAT, ING and GON adipose tissue at the time of euthanization (n=7). **(I)** Taqman-PCR analysis of miR-27a and miR-27b expression in ING adipose tissue isolated from chow and HFD mice. **(J)** qRT-PCR analysis of thermogenesis and adipogenesis associated genes in ING adipose tissue from chow and HFD mice. **(K)** Western blot analysis of UCP1 expression in ING adipose tissue from chow and HFD mice. Data are shown in mean ±SEM, *p<0.05, **p<0.01, ***p<0.001 and ****p<0.0001.

To investigate the impact of obesity development on adipose tissue physiology, we employed a diet-induced obesity (DIO) model using 3-week-old male C57BL/6J mice (Fig. 1A). Mice were fed either a standard control diet or a high-fat diet (HFD) for 16 weeks. HFD-fed mice exhibited a significant increase in body weight compared to control mice (Fig. 1B). After 16 weeks of dietary intervention, HFD mice developed hyperglycemia, as evidenced by elevated fasting glucose levels (Fig. 1C and D). Glucose tolerance tests (GTT) and insulin tolerance tests (ITT) further confirmed significant impairments in both glucose clearance and insulin sensitivity in HFD-fed mice relative to controls (Fig. 1E and F). Following euthanasia at the end of the feeding period, we assessed the weights of various adipose tissue depots, including brown adipose tissue (BAT), subcutaneous (inguinal), and gonadal white adipose tissue (WAT) (Fig. 1G and H). A significant increase in adiposity was observed across all adipose depots in HFD-fed mice, indicating robust obesity development.

Building on our previous findings, where induction of thermogenesis correlated with reduced miR-27a and miR-27b expression in adipose tissue, we examined the expression of these microRNAs in this DIO model. Consistent with earlier observations, miR-27a and miR-27b were significantly downregulated in the inguinal WAT of HFD-fed mice compared to control-fed mice (Fig. 1I). This downregulation was paralleled by a concomitant decrease in the expression of key thermogenic and adipogenic regulators, including *Ucp1, Pparγ, Pgc1α*, and *Prdm16* (Fig. 1J). Western blot analysis in the white adipose tissue also shown decreased UCP1 expression in the HFD mice as compared to control mice (Fig. 1K). The inverse relationship between miR-27a/b expression and the expression of thermogenic genes suggests a potential regulatory axis wherein miR-27a/b levels may modulate the thermogenic and adipogenic capacity of adipose tissue. These findings highlight the potential of miR-27a/b as regulators involved in adipose tissue remodeling in response to obesogenic conditions.

### Combined Deletion of miR-27a and miR-27b Enhances Thermogenic and Adipogenic Potential in Adipocytes:

miR-27a has been shown to bind the 3′-untranslated region (3′-UTR) of PPARγ, suppressing its expression and thereby inhibiting adipocyte differentiation, with an inverse correlation between miR-27a levels and adipogenesis^26^. Similarly, miR-27b suppresses both PPARγ and C/EBPα in human multipotent adipose-derived stem (hMADS) cells, inhibiting lipid accumulation and the expression of late-stage adipogenic markers^27^. Both miR-27a and miR-27b also target prohibitin (PHB), a key upstream regulator of mitochondrial integrity and adipocyte differentiation^18^. Collectively, these miRNAs impair mitochondrial function, suppress browning of white adipose tissue (WAT), and contribute to obesity and insulin resistance.

**Figure 2:**
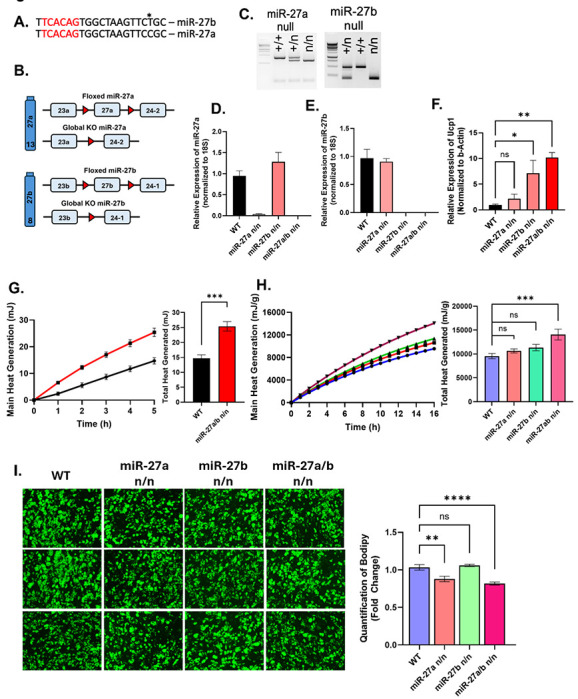
Deletion of miR-27a/b Enhances Thermogenic and Adipogenic Potential in Adipocytes. **(A)** Mature miRNA sequence of miR-27a and miR-27b. (**B)** Schematic representation of strategy used to generate miR-27a and miR-27b knockout mice. **(C)** Representative genotyping images of miR-27a and miR-27b global null, heterozygous and WT mice. **(D and E)** Taqman-PCR for miR-27a and miR-27b expression in the cultured beige adipocytes isolated from stromal vascular fraction (SVF) of inguinal adipose tissue of WT, miR-27a n/n, miR-27b and miR-27a/b null (n=4). **(F)** qRT-PCR for *Ucp1* expression in the cultured beige adipocytes on 10^th^ day of differentiation (n=4). **(G)** Microcalorimetric analysis for heat generation from cultured beige adipocytes isolated from stromal vascular fraction (SVF) of inguinal adipose tissue of WT and miR-27a/b null (n=5). **(H)** Microcalorimetric analysis for heat generation from inguinal adipose tissue isolated from WT, miR-27a n/n, miR-27b and miR-27a/b null 8-week-old mice (n=7). **(I)** BODIPY analysis for lipid accumulation in cultured beige adipocytes on 10^th^ day of differentiation. Data are shown in mean±SEM, *p<0.05, **p<0.01, ***p<0.001 and ****p<0.0001.

miR-27a and miR-27b share an identical seed sequence and differ by only a single nucleotide in their mature sequence (Fig. 2A). While previous studies have explored the individual roles of miR-27a and miR-27b, the combined effect of their deletion on adipose tissue thermogenesis and adipogenesis remains unexplored. To address this gap, we generated global miR-27a, miR-27b, and double knockout (miR-27a/b) mouse models, Fig. 2B shown the schematic representation for generation of knockout mice models. Genotyping and TaqMan PCR has quantitated the expression of miR-27a and miR-27b in the cultured beige adipocytes isolated from the stromal vascular fraction of inguinal adipose tissue from WT and knockout mice models (Fig. 2C–E). *In vitro*, deletion of both miRNAs in cultured beige adipocytes produced a more robust *Ucp1* (uncoupling protein 1) expression response compared to single knockouts or wild-type (WT) cells (Fig. 2F). UCP1 expression is associated with the non-shivering thermogenesis and energy expenditure in brown and beige adipose tissue^28^. This was evident from elevated expression of thermogenic genes

Microcalorimetric analysis of cultured beige and brown adipocytes from the double knockout mice demonstrated significantly increased heat production compared to WT and single knockout counterparts (Fig. 2G, Supp. Fig.1A). To determine whether this phenotype is preserved *in vivo*, we performed microcalorimetry on WAT and BAT isolated from WT, miR-27a, miR-27b, and miR-27a/b mice. Consistent with in vitro findings, double knockout adipose tissues generated significantly more heat than those from single knockouts (Fig. 2H, Supp. Fig.1B). Furthermore, BODIPY staining revealed decreased lipid accumulation in beige adipocytes derived from the double knockout mice compared to single knockouts and WT cells (Fig. 2I), indicating reduced lipid storage capacity and enhanced thermogenic activity.

These findings demonstrate that simultaneous deletion of miR-27a and miR-27b elicits a synergistic effect, enhancing thermogenic gene expression, mitochondrial activity, and lipid metabolism in adipocytes. This provides strong evidence that miR-27a/b acts cooperatively to regulate the thermogenic capacity of white adipose tissue.

### Deletion of miR-27a/b Rescues Browning Capacity and Improves Metabolic Health in HFD-Induced Obese Mice:

**Figure 3:**
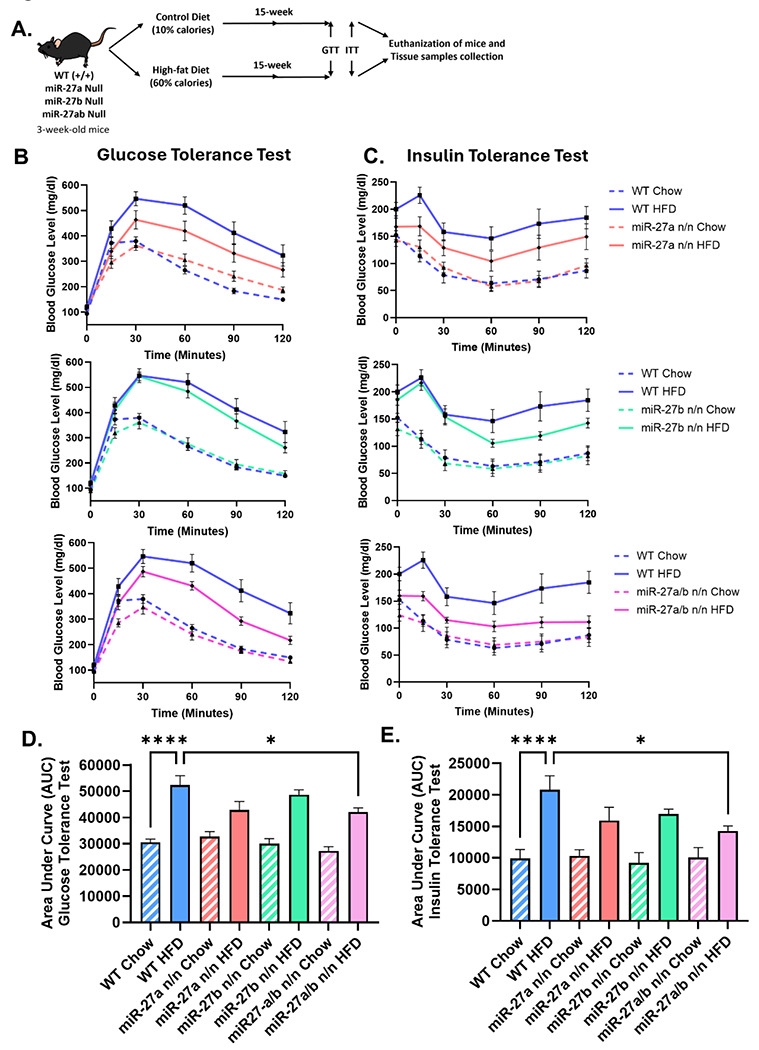
Deletion of miR-27a/b Improves Metabolic Health in HFD Mice. **(A)** Schematic representation of diet induced obesity induction in WT, miR-27a n/n, miR-27b and miR-27a/b null mice. **(B)** Glucose tolerance test of WT, miR-27a n/n, miR-27b and miR-27a/b null male mice after 12h fasting (n=9). **(C)** Insulin tolerance test WT, miR-27a n/n, miR-27b and miR-27a/b null male mice after 6h fasting (n=9). Area under curve analysis of GTT **(D)** and ITT **(E)** of WT, miR-27a n/n, miR-27b and miR-27a/b null mice, respectively (n=9). Data are shown in mean ±SEM, *p<0.05, **p<0.01, ***p<0.001 and ****p<0.0001.

Given the inverse expression pattern of miR-27a/b and Ucp1 observed during high-fat diet (HFD) exposure, we hypothesized that miR-27a/b may contribute to impaired browning capacity of white adipose tissue (WAT) in diet-induced obesity. To assess whether loss of miR-27a/b could rescue thermogenic function, we subjected 3-week-old male wildtype (WT), miR-27a, miR-27b, and miR-27a/b knockout mice to control or HFD feeding for 16 weeks (Fig. 3A). Glucose tolerance tests (GTT) and insulin tolerance tests (ITT) demonstrated enhanced metabolic function in all knockout groups, with double knockout mice showing the most pronounced improvement (Fig. 3B and C). Quantification of the area under the curve (AUC) for both GTT and ITT further confirmed significantly greater glucose and insulin sensitivity in double knockout mice compared to single knockouts (Fig. 3D and E).

**Figure 4:**
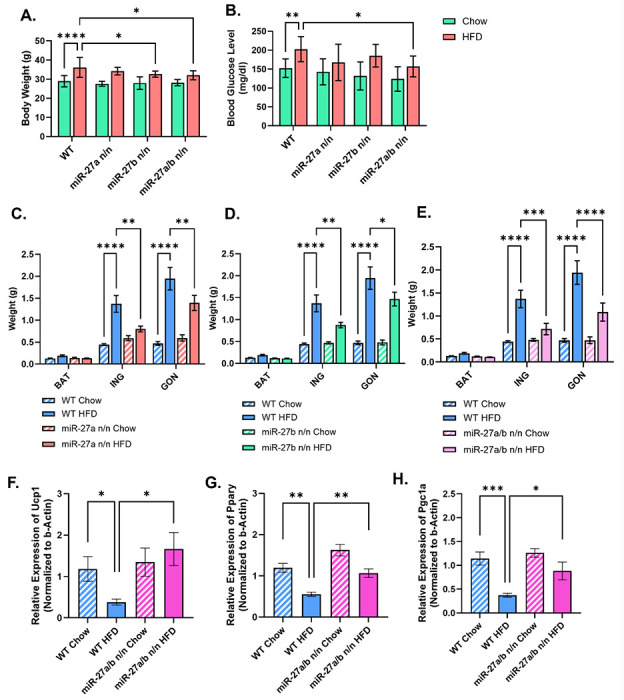
Deletion of miR-27a/b Rescues Browning Capacity in HFD Mice. **(A)** Body weight of mice after 16-weeks of high-fat diet intervention (n=9). **(B)** Blood glucose level of mice after 6h fasting at end of high-fat diet intervention (n=9). **(C)** Weight of BAT, ING and GON tissue weight isolated from WT and miR-27a null mice at the end of HFD diet intervention (n=9). **(D)** Weight of BAT, ING and GON tissue weight isolated from WT and miR-27b null mice at the end of HFD diet intervention (n=9). **(E)** Weight of BAT, ING and GON tissue weight isolated from WT and miR-27a/b null mice at the end of HFD diet intervention (n=9). qRT-PCR analysis of *Ucp1* **(F)**, *Ppary* **(G)**and *Pgc1a* **(H)** expression in ING adipose tissue harvested from WT and miR-27a/b null mice (n=7). Data are shown in mean ±SEM, *p<0.05, **p<0.01, ***p<0.001 and ****p<0.0001.

Mice lacking both miR-27a and miR-27b exhibited significantly reduced body weight gain under HFD conditions compared to WT and single knockout mice (Fig. 4A, Supp. Fig.2). Notably, fasting blood glucose levels were markedly lower in HFD-fed double knockout (DKO) mice relative to WT HFD mice (Fig. 4B), suggesting improved glucose homeostasis. After the 16-week dietary intervention, mice were euthanized, and brown, subcutaneous (inguinal), and gonadal adipose depots were dissected and weighed. In particular, subcutaneous and gonadal WAT from double knockout mice displayed a significant reduction in fat mass, indicative of decreased lipid accumulation and attenuated adiposity (Fig. 4C–E).

Gene expression analysis further revealed a significant upregulation of thermogenic markers, including *Ucp1, Pgc1α*, and *Ppary*, in WAT from HFD-fed DKO mice compared to WT HFD mice (Fig. 4F–H). These findings indicate that the combined deletion of miR-27a and miR-27b enhances thermogenic gene expression and WAT browning, even under obesogenic conditions. Taken together, these data demonstrate that miR-27a/b act cooperatively to suppress thermogenesis and promote adiposity in the context of HFD-induced obesity. Their dual deletion results in more potent metabolic protection and browning potential compared to single miRNA knockouts.

## Discussion

Obesity is a multifactorial metabolic disorder characterized by excessive lipid accumulation, insulin resistance, and impaired energy homeostasis. A growing body of evidence highlights the central role of adipose tissue plasticity, particularly the ability of white adipose tissue (WAT) to acquire thermogenic characteristics, in maintaining metabolic health and counteracting obesity related complications^10, 29, 30^. In this study, we identify miR-27a and miR-27b as critical regulators of adipose tissue thermogenesis and adipogenesis, and we demonstrate that their combined deletion restores metabolic homeostasis and enhances thermogenic potential in the setting of high-fat diet (HFD)-induced obesity.

Our diet-induced obesity (DIO) model recapitulated the hallmarks of metabolic dysfunction, including increased adiposity, hyperglycemia, and insulin resistance, consistent with previous reports^31, 32^. Importantly, we observed a significant downregulation of miR-27a and miR-27b in inguinal WAT of HFD-fed mice. These changes were associated with reduced expression of thermogenic genes, including *Ucp1, Pparγ, Pgc1α*, and *Prdm16*, indicating that prolonged HFD exposure impairs adipose tissue’s capacity to activate thermogenic programs. miR-27a and miR-27b are known to target key transcriptional regulators of adipogenesis, such as *Pparγ*, C/EBPα, and PHB^18, 26, 27^. Previous studies have shown that overexpression of miR-27a suppresses adipocyte differentiation through direct repression of *Pparγ*, and similarly, miR-27b impairs lipid accumulation by targeting both *Pparγ* and C/EBPα^26, 27^. Our study extends these findings by demonstrating that simultaneous deletion of both miR-27a and miR-27b leads to a synergistic enhancement of thermogenic gene expression, mitochondrial activity, and heat production in both cultured adipocytes and adipose tissues *in vivo*. This effect is more robust than that observed in single knockout mice, indicating a functional redundancy and cooperative repression of thermogenesis by these two miRNAs.

Notably, BODIPY staining revealed reduced lipid droplet accumulation in beige adipocytes from miR-27a/b double knockout (DKO) mice, further supporting enhanced mitochondrial oxidation and energy expenditure. These findings are consistent with previous work showing that *Ucp1* activation correlates with reduced lipid storage and increased fatty acid oxidation^33, 34^. Along with this, microcalorimetric analyses revealed significantly elevated heat production in DKO adipocytes and adipose tissues, confirming a gain in functional thermogenesis.

Furthermore, under HFD conditions, DKO mice displayed significantly improved glucose tolerance, insulin sensitivity, and reduced fat mass, emphasizing the metabolic benefits of derepressing the thermogenic program. These results, aligned with previous studies, demonstrate that promoting WAT browning can protect against diet-induced obesity and metabolic dysfunction^10, 35^. Interestingly, while miR-27a/b levels were reduced in HFD-fed mice, this endogenous downregulation was insufficient to maintain thermogenic gene expression, suggesting that sustained repression of thermogenic regulators by residual miR-27a/b may contribute to WAT dysfunction during obesity.

Taken together, our findings identify miR-27a and miR-27b as central nodes in the regulation of adipose tissue thermogenic remodeling. While previous work has examined their individual roles, our study provides the first *in vivo* evidence of their cooperative function in suppressing thermogenesis and promoting adiposity. These data support the therapeutic potential of targeting miR-27a/b to enhance energy expenditure and improve metabolic health in obesity.

## Materials and Methods

### miR27a^acb^ and miR27b^acb^ Strains:

All animal experiments were conducted in accordance with the National Institutes of Health Guide for the Care and Use of Laboratory Animals. The development, characterization, and use of miR-27a and miR-27b knockout (KO) mice, along with other mouse strains, were approved by the Institutional Animal Care and Use Committee (IACUC) of the MaineHealth Institute for Research. Genomic DNA was extracted from mouse tissue samples and used for genotyping via polymerase chain reaction (PCR) targeting miR-27a and miR-27b. In knockout mice, PCR amplification yielded products of 313 bp (miR-27a KO) and 174 bp (miR-27b KO), whereas in wild-type (WT) mice, the respective amplicon sizes were 463 bp and 453 bp. PCR products were visualized using a Bio-Rad ChemiDoc imaging system.

### Strain Maintenance:

Homozygous C57BL/6-miR-27a^acb/acb^ and C57BL/6-miR-27b^acb/acb^ males were bred with C57BL/6J wild-type females to generate heterozygous offspring. These heterozygous mice were then intercrossed to produce experimental cohorts of both male and female mice that were homozygous (acb/acb) or wild type (+/+) for each respective mutation.

To generate miR-27a/b double knockout mice, C57BL/6-miR-27a^acb/acb^ (homozygous miR-27a KO) mice were crossed with C57BL/6-miR-27b^acb/acb^ (homozygous miR-27b KO) mice to produce double heterozygous offspring (C57BL/6-miR-27a^acb/+^, miR-27b^acb/+^). These double heterozygotes were intercrossed to generate experimental cohorts of males and females that were homozygous for both alleles (miR-27a^acb/acb^, miR-27b^acb/acb^) and wild-type (+/+, +/+).

All mice were group-housed in an Association for Assessment and Accreditation of Laboratory Animal Care (AAALAC)–accredited, pathogen-free mouse barrier facility and maintained under a 12-hour light/dark cycle with food and water provided ad libitum.

### Colony Maintenance:

Heterozygous miR-27a, miR-27b, and miR-27a/b male and female mice were intercrossed to generate offspring encompassing all possible genotypes. Litters from these breeding pairs were subsequently used in mating schemes that included wild-type × wild-type, homozygous × homozygous, and heterozygous × heterozygous pairings to establish and maintain the experimental lines.

### Animal Experiments:

For microcalorimetric analysis, brown adipose tissue (BAT) and inguinal white adipose tissue (iWAT) were collected from 8-week-old wild-type (WT), miR-27a^acb/acb^, miR-27b^acb/acb^, and miR-27a/b^acb/acb^ male mice. Tissues were incubated in a CalScreener (Symcel) for 16 hours in DMEM supplemented with 1% bovine serum albumin (BSA), 1× GlutaMAX (Gibco), and 1× antibiotic-antimycotic solution (Gibco).

To establish a high-fat diet (HFD) model, 3-week-old WT, miR-27a^acb/acb^, miR-27b^acb/acb^, and miR-27a/b^acb/acb^ male and female mice were fed either a control diet (10% kcal from fat; Teklad, Cat# TD.08806) or a high-fat diet (60% kcal from fat; Teklad, Cat# TD.06414) for 15 weeks. After 15 weeks of diet intervention, glucose tolerance tests (GTT) and insulin tolerance tests (ITT) were performed. Mice were euthanized 48 hours after ITT, and blood serum and tissues were collected for downstream analyses.

### *In-vitro* Adipocyte Differentiation and Forskolin Treatment:

Preadipocytes were isolated from the brown adipose tissue (BAT) and inguinal white adipose tissue (iWAT) of 8-week-old male wildtype (WT), miR-27a^acb/acb^, miR-27b^acb/acb^, and miR-27a/b^acb/acb^ mice. Dissected tissues were digested in tissue lysis buffer containing 0.123 M NaCl, 1.3 mM CaCl_2_, 5 mM glucose, 100 mM HEPES, 4% (v/v) BSA (fraction V), and 0.1% (w/v) Collagenase P at 37 °C for 30–40 minutes with constant rocking. Following complete digestion, the lysate was filtered through a 70 μm strainer along with an equal volume of autoMACS^®^ Running Buffer to minimize cell clumping. The filtrate was centrifuged at 1200g for 5 minutes, and the resulting pellet was washed with 1× PBS and centrifuged again at 500g for 5 minutes. The final cell pellet was resuspended in DMEM supplemented with 10% fetal bovine serum (FBS), 1× GlutaMAX, and 1× antibiotic-antimycotic solution, and plated in 10 cm or 15 cm culture dishes based on cell yield.

For differentiation, preadipocytes were seeded into 12-well or 24-well plates in growth medium (DMEM with 10% FBS, 1x GlutaMAX, and 1x Anti-Anti). Upon reaching 100% confluency, cells were incubated for 3 days in differentiation medium composed of DMEM supplemented with 1.7 mM insulin, 1 μM T3, 5μM rosiglitazone, 10 μM SB431542, 500 μM IBMX, 10 μM dexamethasone, 125 μM indomethacin, and 50 μg/mL L-ascorbic acid 2-phosphate (AA2P). After 3 days, the medium was replaced with maintenance medium (DMEM containing 1.7 mM insulin, 1 μM T3, 5 μM rosiglitazone, 10 μM SB431542, and 50 μg/mL AA2P) and incubated for another 3 days. Thereafter, the cells were cultured in regular growth medium (DMEM + 10% FBS, 1x GlutaMAX, and 1x Anti-Anti) until day 10 of differentiation.

For forskolin treatment, mature adipocytes were serum-starved overnight in DMEM containing 1% BSA, 1x GlutaMAX, and 1x Anti-Anti, on 9^th^ day of differentiation. The following day, cells were treated with 10 μM forskolin (FSK) for 6 hours in serum-starved medium. After treatment, cells were harvested for downstream analysis. RNA and protein were extracted using QIAzol and RIPA buffer, respectively, to assess mRNA expression and protein levels.

### Glucose and Insulin Tolerance Tests (GTT and ITT):

For glucose tolerance tests (GTT), mice fed either control or high-fat diet were fasted for 12 hours. Baseline fasting blood glucose levels were measured prior to intraperitoneal injection of glucose (2 g/kg body weight). Blood glucose levels were subsequently measured at 15, 30, 60, 90, and 120 minutes post-injection. For insulin tolerance tests (ITT), mice were fasted for 6 hours, and baseline blood glucose was measured before intraperitoneal administration of insulin (0.5 IU/kg body weight). Blood glucose levels were similarly recorded at 15, 30, 60, 90, and 120 minutes after insulin injection. All blood glucose measurements were performed using the Accu-Chek Guide Me glucometer (Roche Diagnostics).

### Serum and Tissue Sample Processing:

At the end of the high-fat diet (HFD) study, mice were euthanized, and blood was collected via cardiac puncture. Serum was isolated by centrifugation at 1300g for 10 minutes at 4°C, then aliquoted and stored at −80°C for subsequent analyses. Tissue samples for RNA extraction were homogenized in Qiazol using a Bead Beater homogenizer and stored at −80°C until further processing. Protein samples were snap-frozen in liquid nitrogen and stored at −80°C for later use. Tissues designated for histological analysis were fixed in 10% neutral-buffered formalin overnight at 4°C, followed by paraffin embedding, sectioning, and processing for routine histology or immunostaining.

### RNA Isolation and Quantitative PCR:

Total RNA from cells and tissues was extracted using the miRNeasy Micro Kit (Qiagen) according to the manufacturer’s protocol. RNA concentration and purity were assessed using a NanoDrop^™^ 2000 spectrophotometer (ThermoFisher Scientific), ensuring 260/280 absorbance ratios between 1.9 and 2.0. For mRNA analysis, 300 ng of total RNA was reverse transcribed using the qScript cDNA Synthesis Kit (Quantabio). Quantitative PCR (qPCR) was performed using AzuraView^™^ GreenFast qPCR Blue Mix LR (Azura Genomics) on a CFX Opus 384 Real-Time PCR System (Bio-Rad) with gene-specific primers (listed in Table XX). For microRNA analysis, 20 ng of RNA was reverse transcribed using the TaqMan^™^ MicroRNA Reverse Transcription Kit (ThermoFisher Scientific) along with miRNA-specific TaqMan^™^ primers for miR-27a and miR-27b. qPCR for miRNAs was performed using TaqMan^™^ Fast Universal PCR Master Mix on the same Bio-Rad platform with miRNA-specific TaqMan^™^ probes. β-Actin and 18S rRNA were used as internal controls for normalization of mRNA and miRNA expression, respectively. Relative expression levels were calculated using the ΔΔCt method.

### Protein Isolation and Western Blot Analysis:

Protein lysates were prepared from cells and tissues using RIPA lysis buffer (Sigma-Aldrich) supplemented with phosphatase and protease inhibitor cocktails (Sigma-Aldrich). 20 μg of total protein per sample was separated by SDS-PAGE and transferred to PVDF membranes for immunoblotting. Membranes were incubated with a primary antibody against UCP1 (Cell Signaling Technology; 1:1000 dilution), followed by incubation with an HRP-conjugated secondary antibody (Cell Signaling Technology; 1:5000 dilution). Protein bands were visualized using Clarity^™^ and Clarity Max^™^ ECL Western Blotting Substrates (Bio-Rad). β-Tubulin or β-Actin was used as a loading control for normalization. Densitometric analysis of immunoblots was performed using ImageJ software, and signal intensity was normalized to background levels.

### BODIPY staining:

Preadipocytes were isolated from inguinal adipose of WT, miR-27a, miR-27b and miR-27a/b knockout mice. Cells were cultured and induced to differentiate under standard adipogenic conditions. On day 10 of differentiation, cells were washed with PBS and incubated with BODIPY 493/503 (Invitrogen; diluted 1:2000) for 20 minutes at room temperature to visualize intracellular lipid droplets. After staining, cells were washed and imaged using a fluorescence microscope. BODIPY staining intensity served as a qualitative measure of lipid accumulation and adipocyte differentiation efficiency.

### Heat Production Assay via Microcalorimetry:

Heat output from beige adipocytes and freshly excised adipose tissue was assessed using high-resolution isothermal microcalorimetry with the calScreener^™^ system (Symcel). Beige preadipocytes were cultured and differentiated in DMEM (as described above) within Geltrex-coated calWell^™^ insert plates (Symcel, 1900402), then transferred into screw-cap titanium vials. The calPlate^™^ (48-well format) was equilibrated for 30 minutes across two thermal zones, followed by a 15–30 minute stabilization phase after placement. Heat generation was continuously monitored using calView^™^ software. For *ex vivo* experiments, tissue samples were collected from anatomically matched sites within the fat pad, placed into calWells containing 200 μL of DMEM, and normalized by tissue weight. All readings were adjusted relative to control wells containing media only.

### Statistical Analysis:

All experiments were performed in biological triplicates or more, and data are presented as mean ± standard error of the mean (SEM). Statistical significance was assessed using Student’s *t*-test (for comparisons between two groups) or one-way analysis of variance (ANOVA) followed by appropriate post hoc tests (for comparisons among multiple groups). A p-value < 0.05 was considered statistically significant.

### Animal Ethics:

All animal studies were conducted in compliance with the National Institutes of Health Guide for the Care and Use of Laboratory Animals and were approved by the Institutional Animal Care and Use Committee (IACUC; protocol #2203) at the MaineHealth Institute for Research. Mice were housed in pathogen-free facilities with *ad libitum* access to food and water. Every effort was made to minimize animal suffering and reduce the number of animals used.

### Data Availability:

All data supporting the findings of this study are included in the article and its Supplementary Information. Raw and processed data from RNA and microRNA qPCR screens, microcalorimetry, and metabolic cage studies are available from the corresponding author upon reasonable request.

## Supplementary Material

Supplement 1
