# Supplementary material for: Isoform-Specific Control of Adipose Thermogenesis by the miR-27 Family Reveals Antagonism within a Polycistronic miRNA Cluster
Source: bioRxiv. 2026 Jan 21:2025.07.21.666011. Originally published 2025 Jul 25. Preprint. [Version 2] doi: 10.1101/2025.07.21.666011 (PMC12330670; doi:10.1101/2025.07.21.666011)
Supplement: Supplement 1 [file media-1.pdf]

Supplementary Figure 1

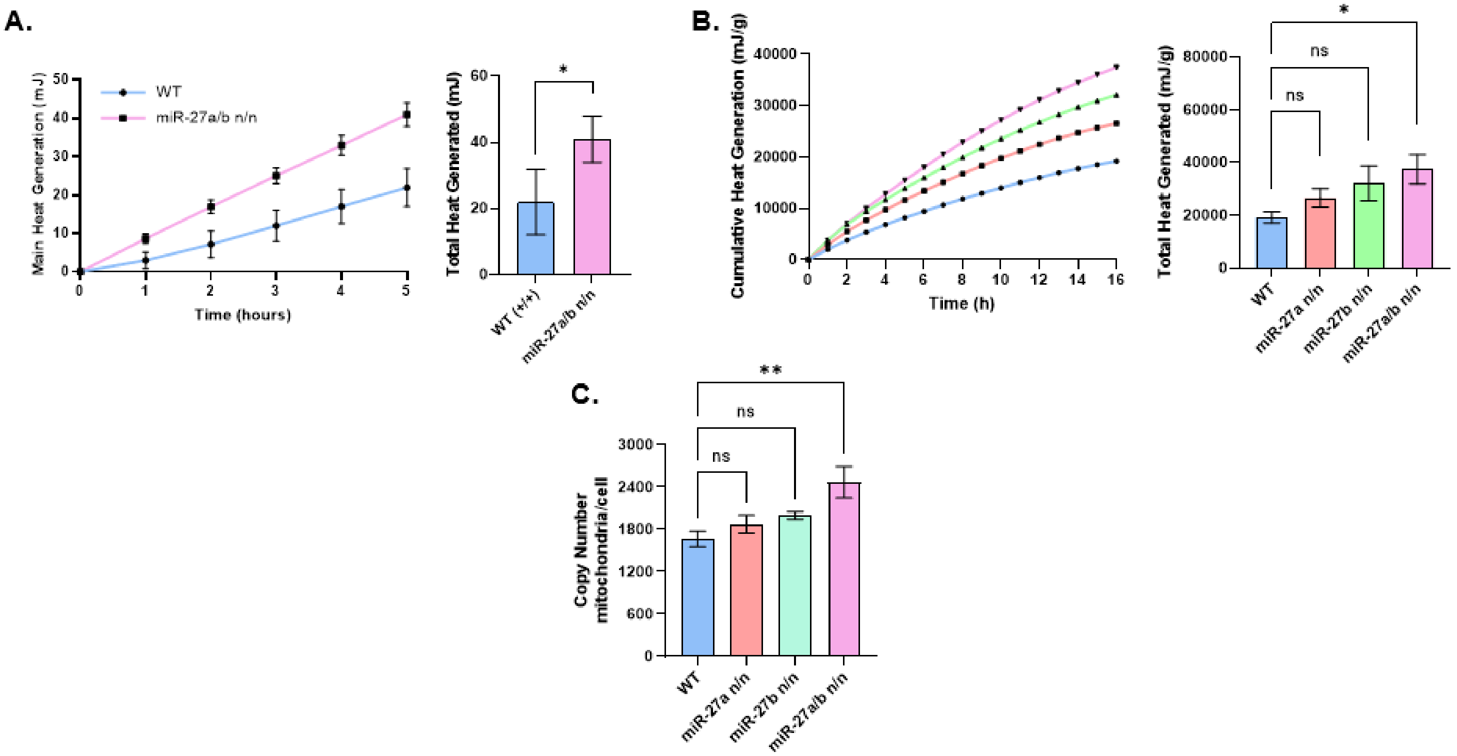

Supplementary Figure 1. Thermogenic function and mitochondrial abundance in brown adipocytes and brown adipose tissue. (A) Isothermal microcalorimetric analysis of heat production from cultured brown adipocytes differentiated from stromal vascular fraction isolated from brown adipose tissue (BAT) of WT and miR-27a/b DKO mice (n = 5). Left panel shows cumulative heat production over time; right panel displays quantification of total heat output derived from time-course measurements. **(B)** Isothermal microcalorimetric analysis of heat generation from BAT explants isolated from 8-week-old WT, miR-27a knockout (KO), miR-27b KO, and miR-27a/b DKO mice (n = 7). Left panel shows cumulative heat production over time; right panel shows quantification of total heat output. **(C)** Mitochondrial DNA copy number analysis in BAT isolated from 8-week-old WT, miR-27a KO, miR-27b KO, and miR-27a/b DKO mice (n = 4). Data are presented as mean ± SEM. Statistical significance is indicated as *p < 0.05, **p < 0.01, and ****p < 0.0001.

Supplementary Figure 2

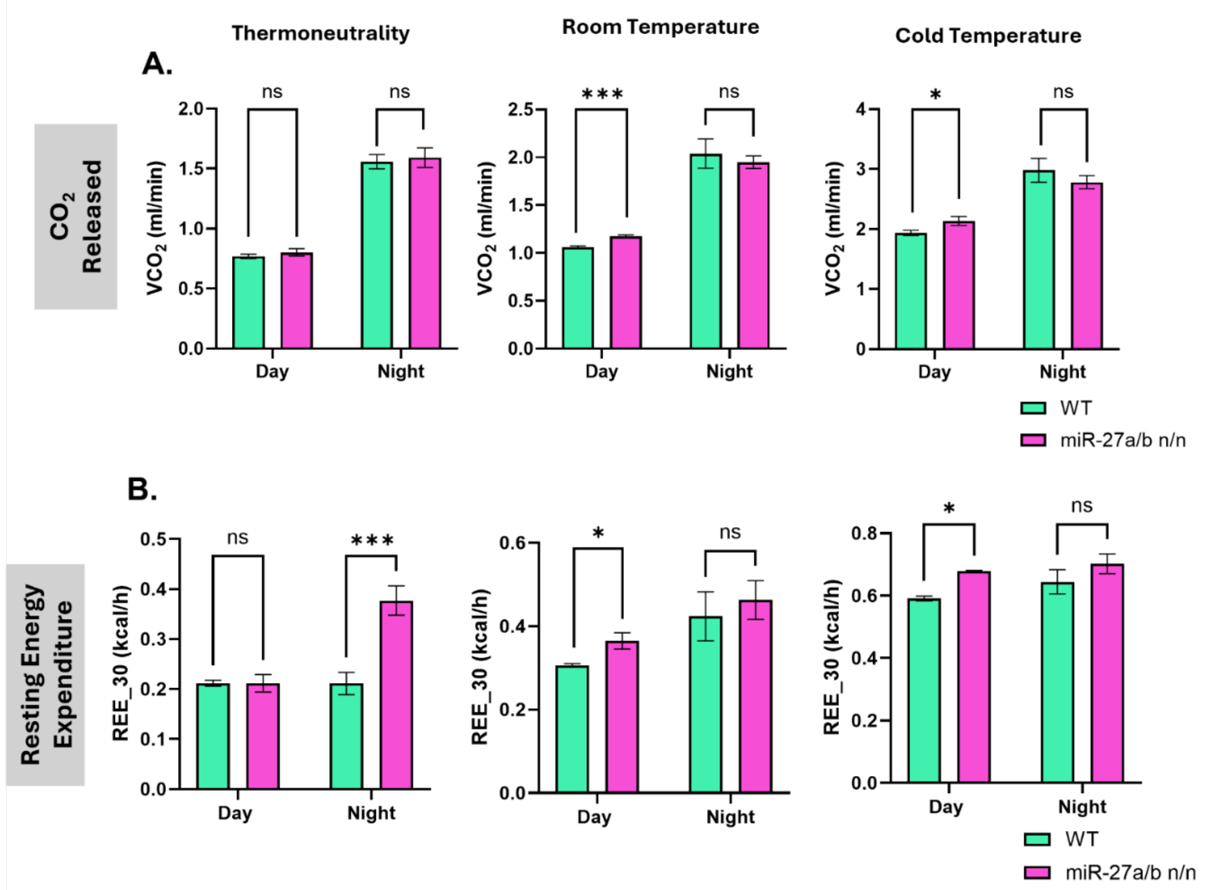

Supplementary Figure 2. Metabolic cage assessment of respiratory output and resting energy expenditure in WT and miR-27a/b DKO mice. (A) Carbon dioxide production (VCO_2) measured in wild-type WT and miR-27a/b DKO mice under thermoneutral (28 °C), room temperature (22 °C), and cold (6.5 °C) conditions. **(B)** Mean resting energy expenditure calculated during the lowest 30-minute activity period (REE_{30}) in WT and DKO mice across the same temperature conditions. Data are presented as mean ± SEM. Statistical significance is indicated as *$p < 0.05$ and ***$p < 0.001$.

Supplementary Figure 3

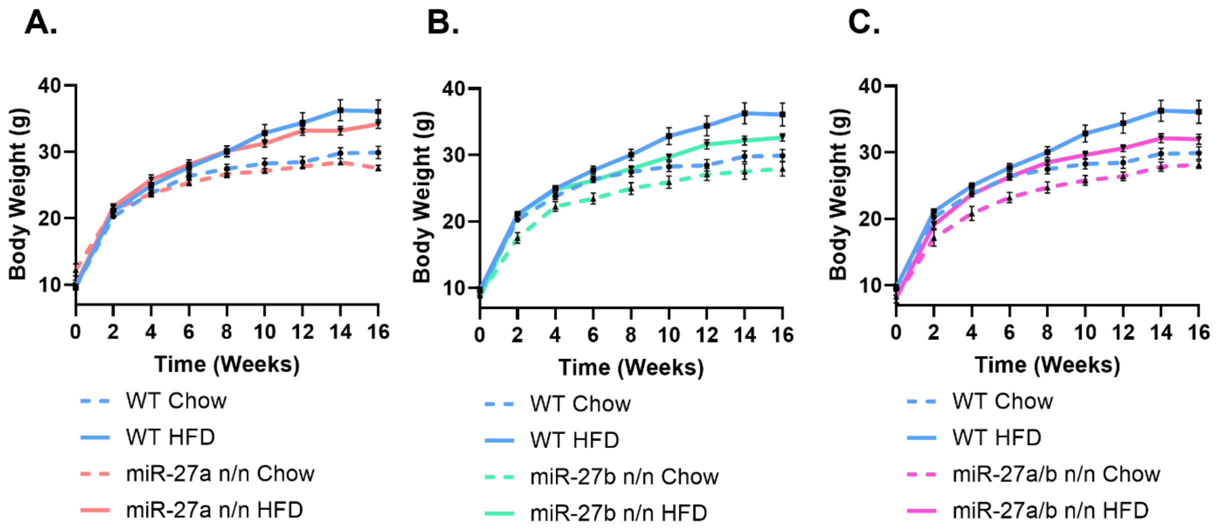

Supplementary Figure 3. Body weight trajectories during chow and high-fat diet feeding in miR-27 knockout mice. (A-C) Longitudinal body weight measurements of male miR-27a KO **(A)**, miR-27b KO **(B)**, and miR-27a/b DKO **(C)** mice maintained on standard chow or high-fat diet (HFD), shown in comparison with WT controls (n = 9 per group). Data are presented as mean ± SEM.
