# Supplementary material for: Isoform-Specific Control of Adipose Thermogenesis by the miR-27 Family Reveals Antagonism within a Polycistronic miRNA Cluster
Source: bioRxiv. 2026 Jan 21:2025.07.21.666011. Originally published 2025 Jul 25. Preprint. [Version 2] doi: 10.1101/2025.07.21.666011 (PMC12330670; doi:10.1101/2025.07.21.666011)
Supplement: Supplement 2 [file NIHPP2025.07.21.666011v2-supplement-2.pdf]

bioRxiv preprint doi: https://doi.org/10.1101/2025.07.21.666011; this version posted January 21, 2026. The copyright holder for this preprint (which was not certified by peer review) is the author/funder, who has granted bioRxiv a license to display the preprint in perpetuity. It is made available under aCC-BY 4.0 International license.

Supplementary Table 1. Breeding performance and sex distribution of miR-27 knockout lines

Genotype	Breeders	Conceived females	Male pups	Female pups	Male ratio	Female ratio	P-value	Fertility (%)	Pups from 1st litter	Mean litter size
WT	9	8	79	69	0.534	0.466	0.946	88.89	49	5.44
miR-27a Null	9	7	58	53	0.523	0.477	0.964	77.78	36	4.0
miR-27b Null	9	7	62	48	0.564	0.436	0.899	77.78	42	4.67
miR-27a/b DKO	10	7	57	49	0.538	0.462	0.94	70.0	32	3.2

Sex-ratio P-values were calculated using chi-square tests against an expected 1:1 male:female ratio. Fertility rate reflects the proportion of breeding females producing litters. Mean litter size was calculated from first litters only. No statistically significant deviations in sex ratio were detected among genotypes ($p > 0.05$).

Supplementary Table 2. Primer sequences used for quantitative PCR

Gene	Forward Primer (5'→3')	Reverse Primer (5'→3')
Ucp1	ACGTCCCCTGCCATTTTACTG	CTTGGGTACTGTCCTGGCAG
Pgc1a	GAATCAAGCCACTACAGACACCG	CATCCCTCTTGAGCCTTTCGTG
Pparγ	GTACTGTCGGTTTCAGAAGTGCC	ATCTCCGCCAACAGCTTCTCCT
Prdm16	ATCCACAGCAGCGGTGAAGCCAT	ACATCTGCCCACAGTCCTTGCA
Cox7a1	CAGCGTCATGGTCAGTCTGT	AGAAAACCGTGTGGGCAGAGA
Cox8b	GAACCATGAAGCCAACGACT	GCGAAGTTCACAGTGGTTCC
Cidea	GGTGGACACAGAGGAGTTCTTTC	CGAAGGTGACTCTGGCTATTCC
Actb (β-actin)	CATTGCTGACAGGATGCAGAAGG	TGCTGGAAGGTGGACAGTGAGG
Aopep	GGAGCTGATGGTGAGTGAGG	TTGATGTCCAGGGTCAGTGC
Fancc	CGCAAAATCCCTTTGAGAGC	CTGTCAAGCTCTGGGTCTGG
miR-23a	ATCACATTGCCAGGGATTTCC	GAATCGAGCACCAGTTACGC
miR-24-2	GTGCCTACTGAGCTGAAACAGT	CTGTTCCGTGCTGAACTGAGCCA
miR-23b	ATCACATTGCCAGGGATTACCAC	GAATCGAGCACCAGTTACGC
miR-24-1	GTGCCTACTGAGCTGATATCAGT	ACTGATATCAGCTCAGTAGGCAC
