# Supplementary material for: Combined Deletion of miR-27a and miR-27b Enhances Protection Against Diet-Induced Obesity
Source: bioRxiv. 2025 Jul 25:2025.07.21.666011. Preprint. [Version 1] doi: 10.1101/2025.07.21.666011 (PMC12330670; doi:10.1101/2025.07.21.666011)
Supplement: Supplementary file 1 [file media-1.pdf]

Supplementary Figure 1

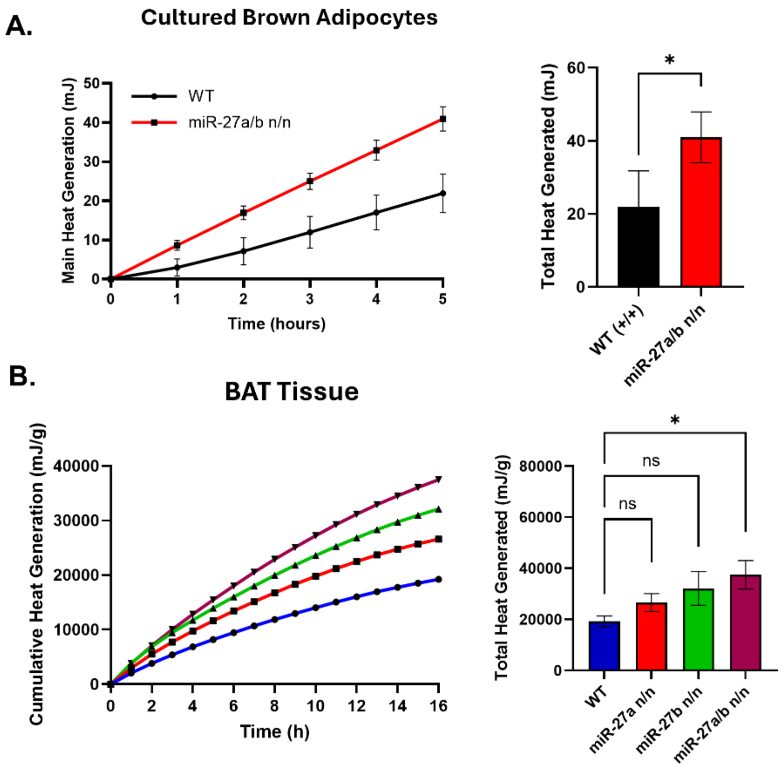

Supplementary Figure 1: (A) Microcalorimetric analysis for heat generation from cultured beige adipocytes isolated from stromal vascular fraction (SVF) of brown adipose tissue of WT and miR-27a/b null (n=5). **(B)** Microcalorimetric analysis for heat generation from Brown adipose tissue isolated from WT, miR-27a n/n, miR-27b and miR-27a/b null 8-week-old mice (n=7). Data are shown in mean ±SEM, *p<0.05.

Supplementary Figure 2

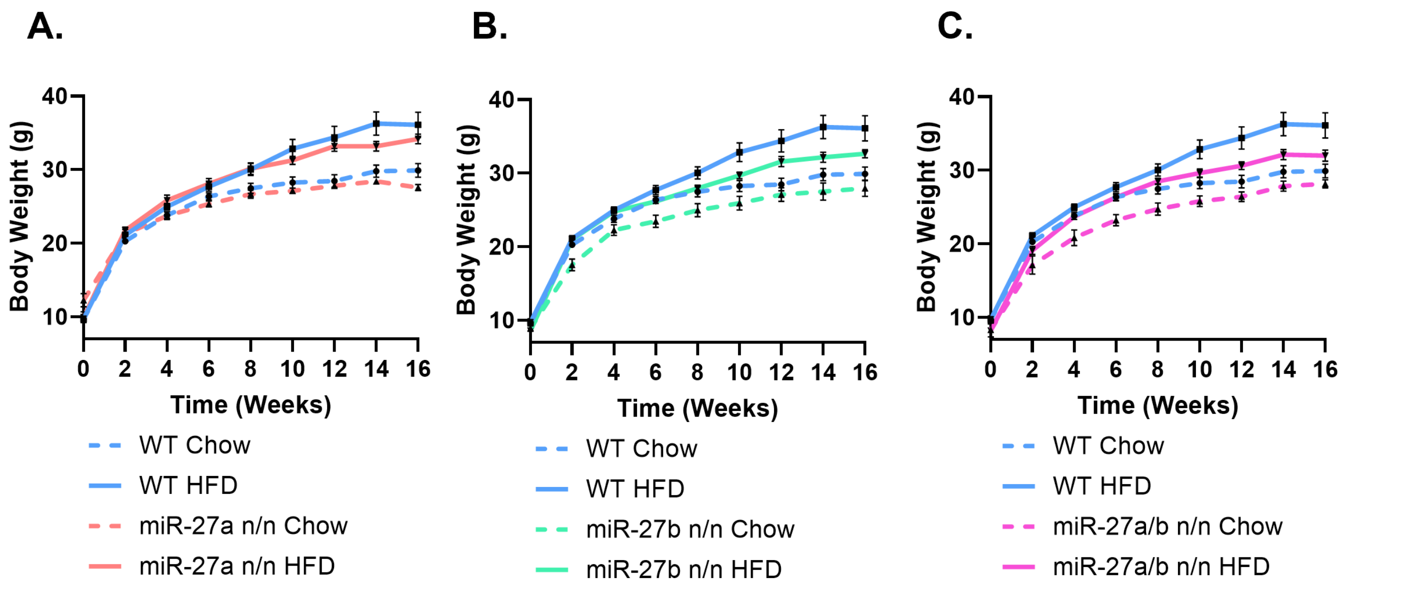

Supplementary Figure 2: Body weight of miR-27a n/n **(A)**, miR-27b **(B)** and miR-27a/b **(C)** null male mice during high-fat diet intervention in compared to WT mice (n=9). Data are shown in mean ±SEM.
